# Supplementary material for: Identification of an orally active small-molecule PTHR1 agonist for the treatment of hypoparathyroidism
Source: Nat Commun. 2016 Nov 18;7:13384. doi: 10.1038/ncomms13384 (PMC5120204; doi:10.1038/ncomms13384)
Supplement: Supplementary Information — Supplementary Figures 1-19 and Supplementary Tables 1-4 [file ncomms13384-s1.pdf]

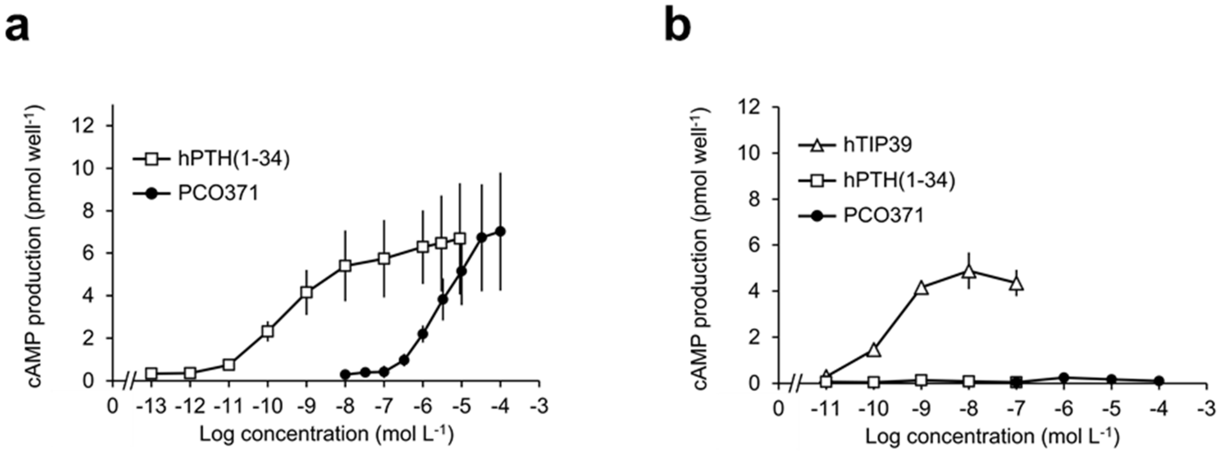

Supplementary Figure 1. Reactivity of PCO371 to rat PTHR1 and PTHR2. (**a**) cAMP production by hPTH(1-34) and PCO371 in COS-7 cells expressing rat PTHR1. (**b**) cAMP production by hPTH(1-34), PCO371, and hTIP39 in COS-7 cells expressing rat PTHR2. Data are represented as the mean ± s.d. of one experiment (*n*=3).

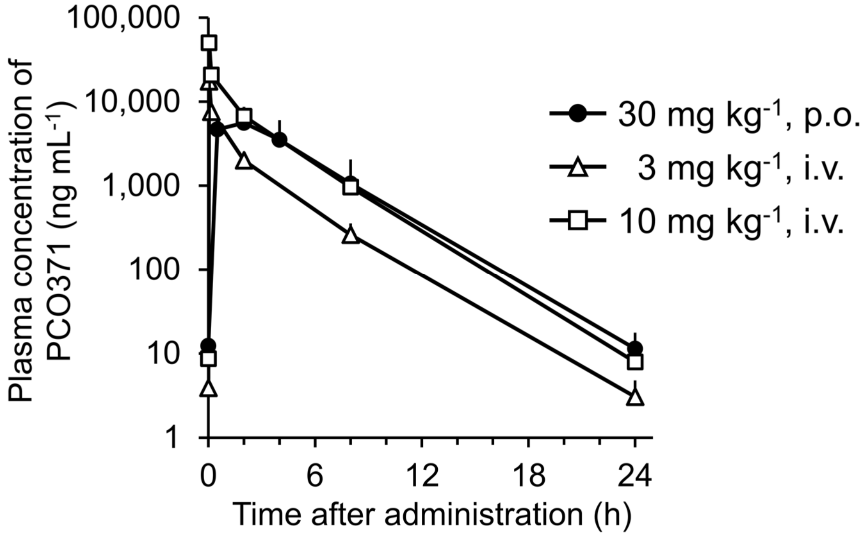

Supplementary Figure 2. Pharmacokinetic profile of PCO371 in OVX rats. OVX rats were treated with a single oral (30 mg kg-1) or intravenous (3 or 10 mg kg-1) administration of PCO371. Data are represented as the mean + s.d. of one experiment (*n*=11 for 3 and 30 mg kg^{-1} PCO371, *n*=12 for 10 mg kg^{-1} PCO371). Additional pharmacokinetics parameters are described in **Supplementary Table 2**.

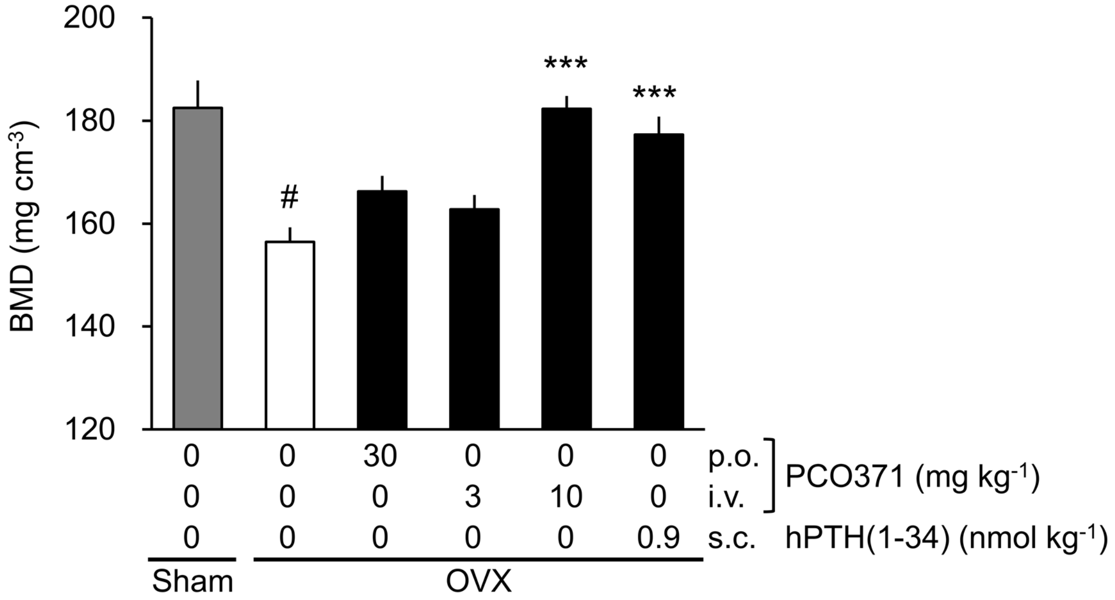

Supplementary Figure 3. BMD of the total femur of OVX rats. Rats were treated once-daily with PCO371 (p.o.) or hPTH(1-34) (s.c.) for 12 weeks commencing at 12 weeks after OVX surgery. Data are represented as the mean + s.e.m. of one experiment [*n*=9 for Sham, *n*=12 for Vehicle and hPTH(1-34), *n*=10 for 10 mg kg^{-1} POC371, *n*=11 for 3 and 30 mg kg^{-1} PCO371). Student's *t*-test was used to compare the sham and OVX vehicle-treated groups; $^{\#}$: P<0.05. Parametric Dunnett's test was used to compare PCO371- or hPTH(1-34)-treated groups with the OVX vehicle-treated group; *: P<0.05, **: P<0.01, ***: P<0.001.

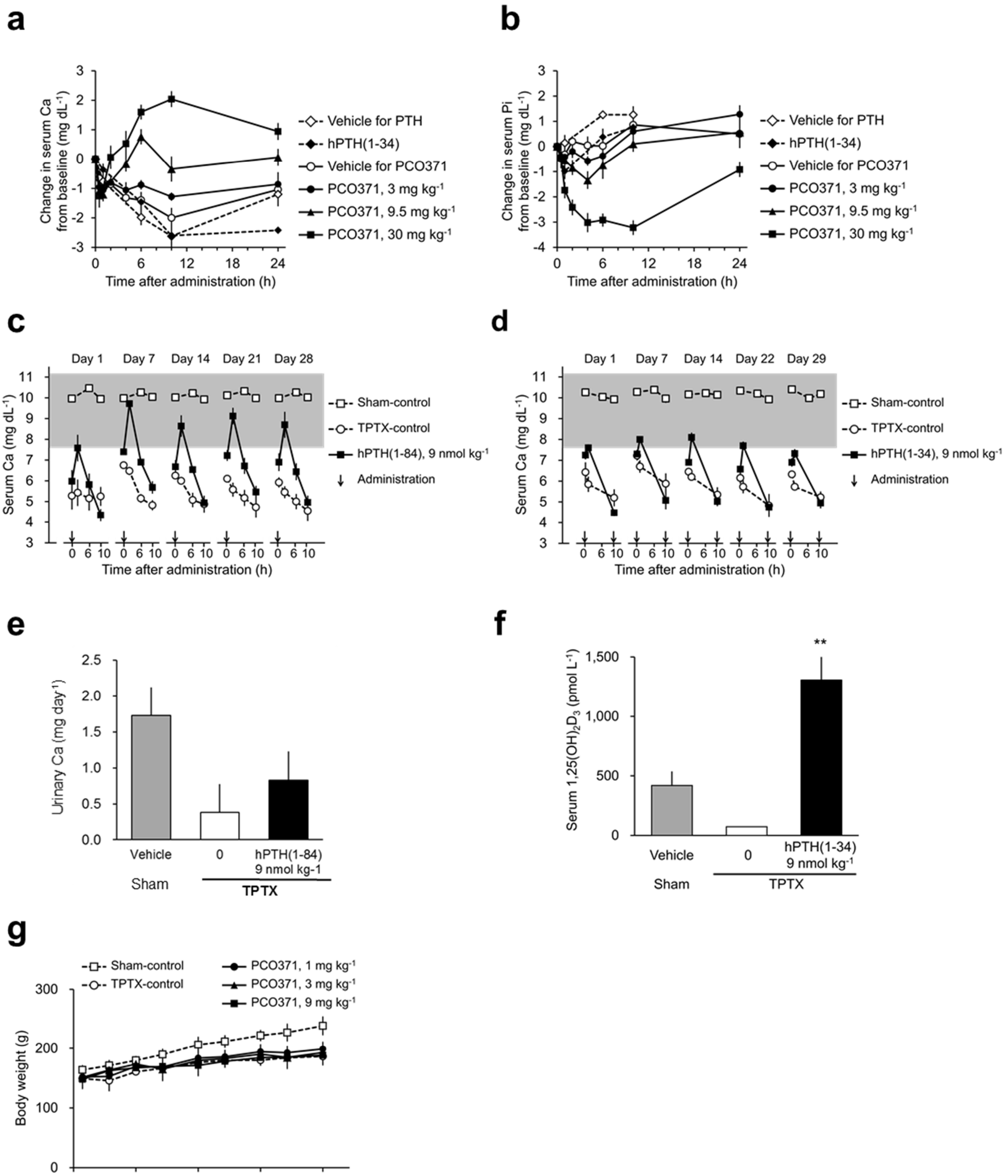
a
Change in serum Ca from baseline (mg dL-1)
Time after administration (h)
Vehicle for PTH
hPTH(1-34)
Vehicle for PCO371
PCO371, 3 mg kg-1
PCO371, 9.5 mg kg-1
PCO371, 30 mg kg-1
b
Change in serum Pi from baseline (mg dL-1)
Time after administration (h)
Vehicle for PTH
hPTH(1-34)
Vehicle for PCO371
PCO371, 3 mg kg-1
PCO371, 9.5 mg kg-1
PCO371, 30 mg kg-1
c
Day 1 Day 7 Day 14 Day 21 Day 28
Serum Ca (mg dL-1)
Time after administration (h)
Sham-control
TPTX-control
hPTH(1-84), 9 nmol kg-1
Administration
d
Day 1 Day 7 Day 14 Day 22 Day 29
Serum Ca (mg dL-1)
Time after administration (h)
Sham-control
TPTX-control
hPTH(1-34), 9 nmol kg-1
Administration
e
Urinary Ca (mg day-1)
Vehicle
0
hPTH(1-84) 9 nmol kg-1
Sham
TPTX
f
Serum 1,25(OH)2D3 (pmol L-1)
**
Vehicle
0
hPTH(1-34) 9 nmol kg-1
Sham
TPTX
g
Sham-control
TPTX-control
PCO371, 1 mg kg-1
PCO371, 3 mg kg-1
PCO371, 9 mg kg-1
Body weight (g)
Treatment time (day)

Supplementary Figure 4. Effects of PCO371 on serum parameters in TPTX rats. (**a,b**) Calcemic (**a**) and hypophosphatemic (**b**) effects of oral PCO371 in single administration or in single twice-daily subcutaneous treatment with hPTH(1–34) (*n*=5 for each dose). (**c,d**) Changes in serum Ca level after once-daily subcutaneous treatment (arrows) with hPTH(1–84) (*n*=5 for Sham and 9 nmol kg^{-1} hPTH(1–84), *n*=4 for TPTX-control) (**c**) and twice-daily subcutaneous treatment (arrows) with hPTH(1–34) (*n*=5 for Sham, *n*=4 for TPTX control and 9 nmol kg^{-1} hPTH(1–34)) (**d**), with shaded areas showing the target therapeutic range (7.6–11.2 mg dL^{-1}) of serum Ca. (**e**) Urinary Ca excretion in TPTX rats after once-daily administration of hPTH(1–84) for 4 weeks (*n*=5 for Sham and 9 nmol kg^{-1} hPTH(1–84), *n*=4 for TPTX control). (**f**) Levels of 1,25$(OH)_2D_3$ in serum after twice-daily administration of hPTH(1–34) for 4 weeks (*n*=5 for Sham, *n*=3 for TPTX control, *n*=4 for 9 nmol kg^{-1} hPTH(1–34)). (**g**) Changes in body weight in TPTX rats treated twice-daily with oral PCO371 for 4 weeks (*n*=5 for each dose). Data are represented as the mean ± s.e.m. of one experiment. Student's t-test was used to compare the TPTX vehicle-treated groups with the hPTH(1–34)-treated group;**: *P*<0.01.

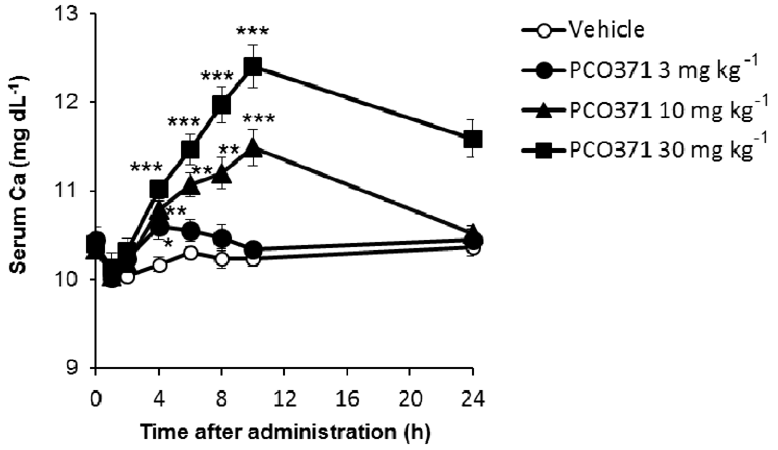

Supplementary Figure 5. Calcemic effect of PCO371 in normal dogs. Time course changes in serum Ca levels in normal dogs after single oral administration of PCO371 (*n*=6 for each dose). Data are represented as the mean ± s.e.m. of one experiment. Dunnett's test was used to compare the vehicle-treated groups with the PCO371-treated groups.*: *P*<0.05, **: *P*<0.01, ***: *P*<0.001.

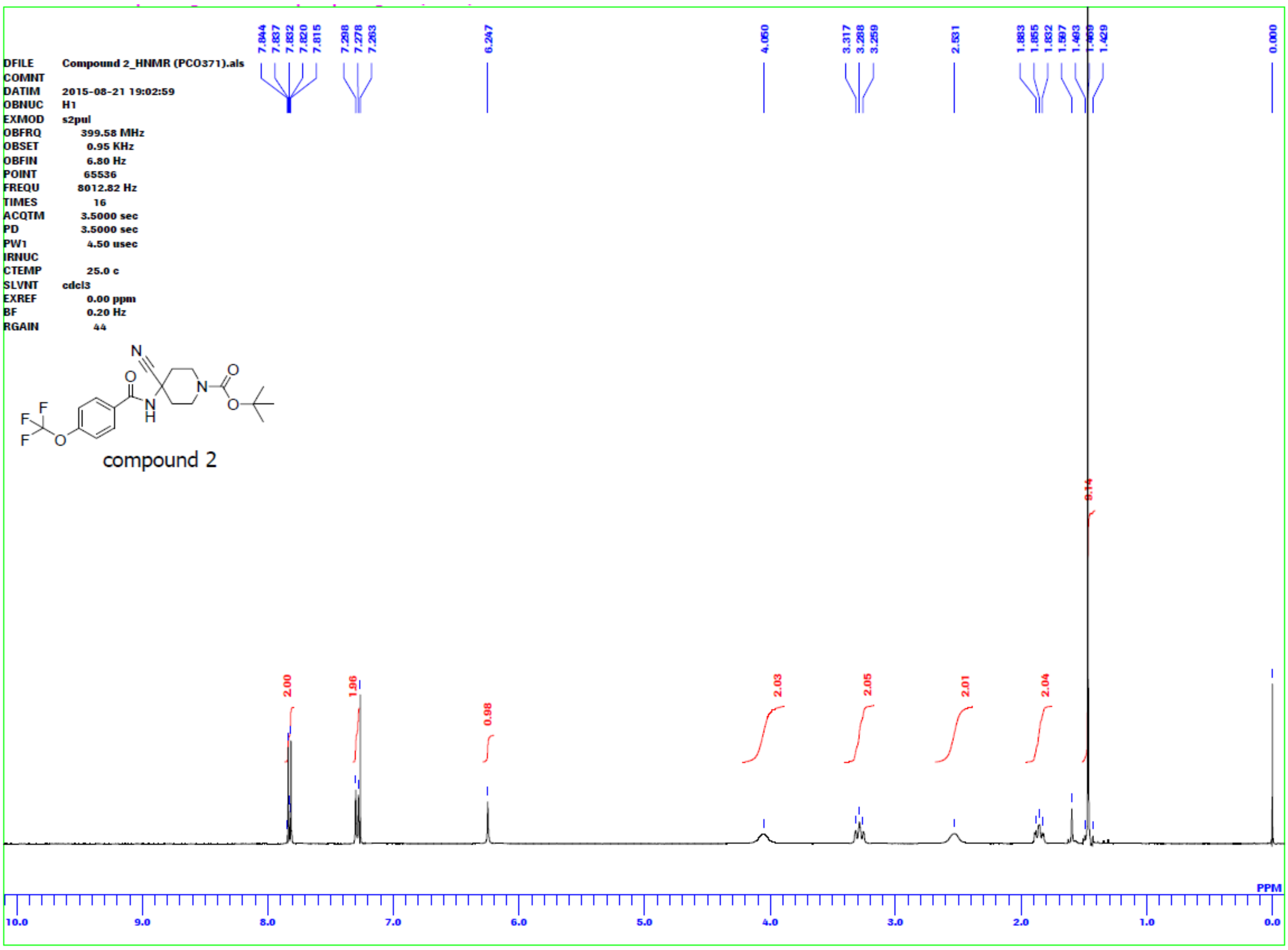

Supplementary Figure 6. **^{1}H-NMR (400 MHz, $CDCl_3$) spectrum of compound 2.**

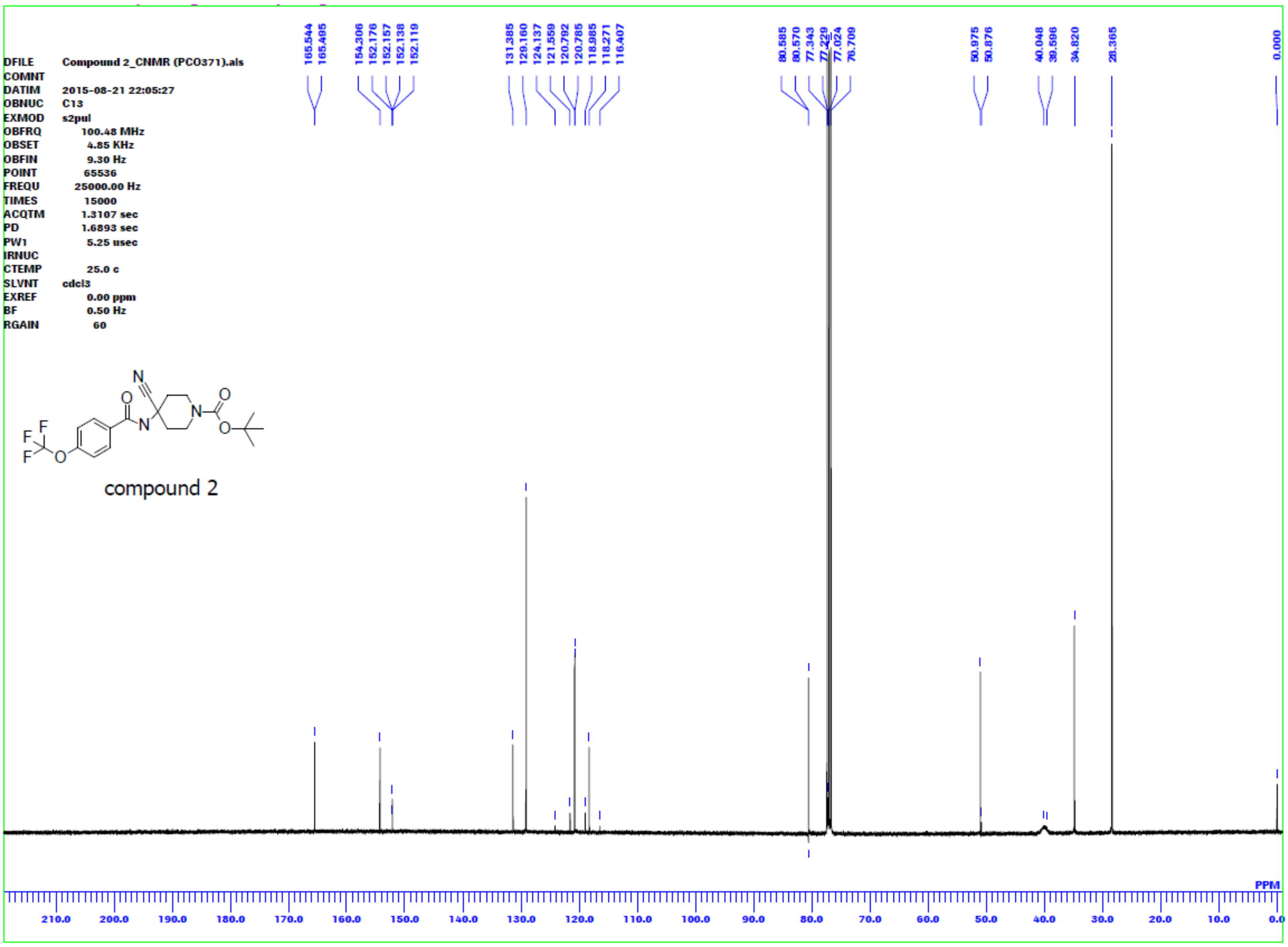

Supplementary Figure 7. **^{13}C-NMR (100 MHz, $CDCl_3$) spectrum of compound 2.**

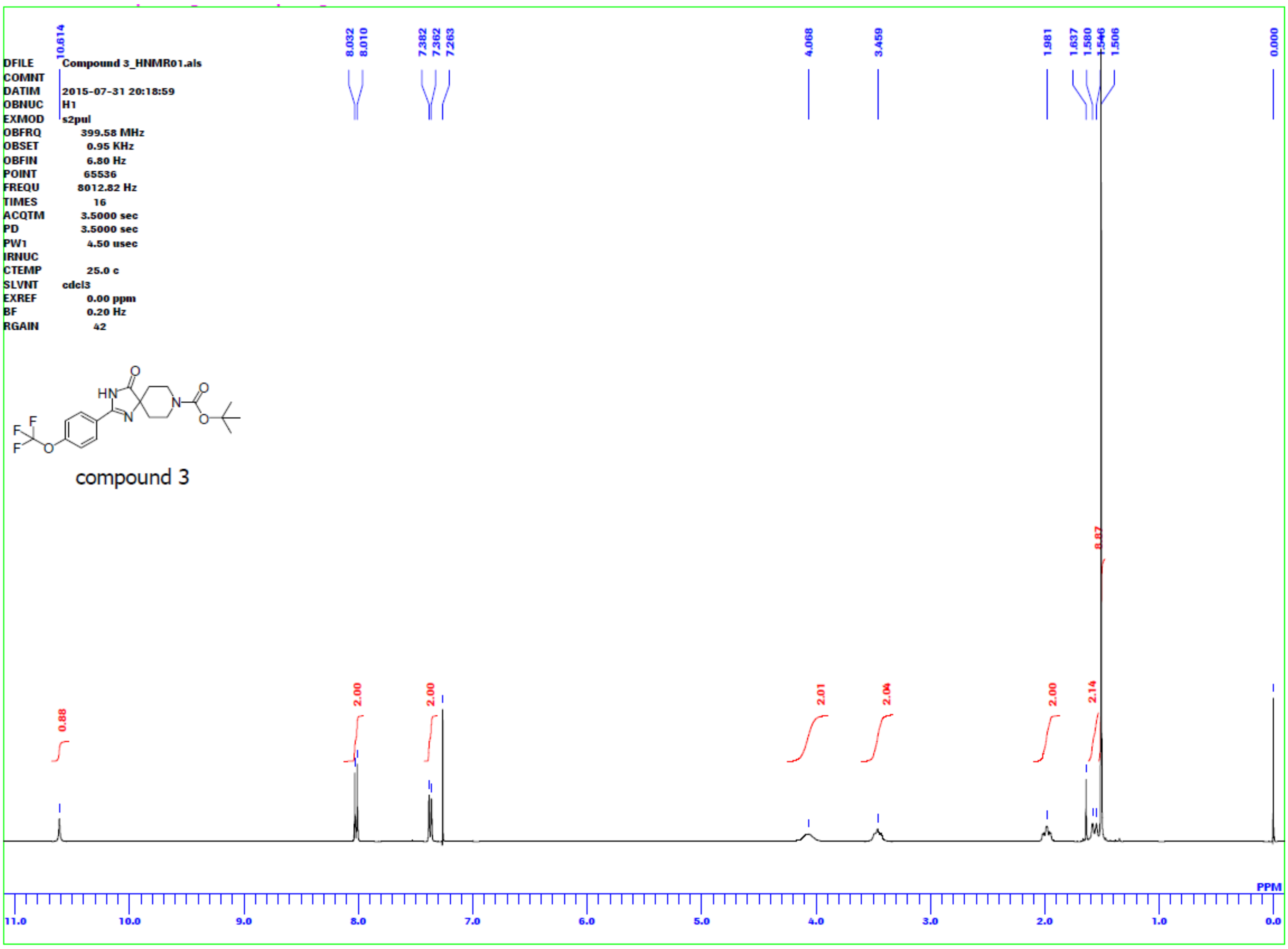

Supplementary Figure 8. ^{1}H-NMR (400 MHz, $CDCl_3$) spectrum of compound 3.

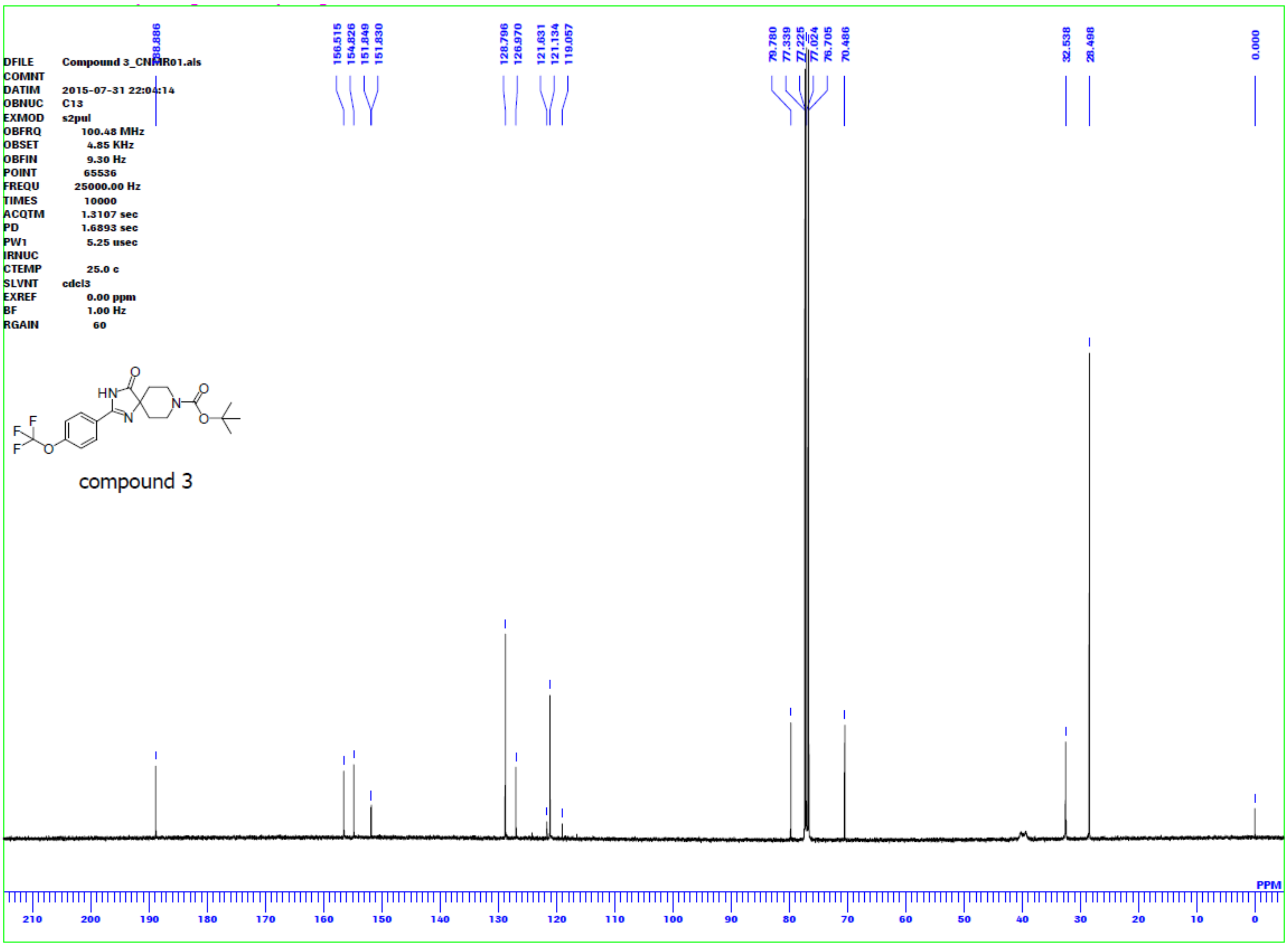

Supplementary Figure 9. ^{13}C-NMR (100 MHz, $CDCl_3$) spectrum of compound 3.

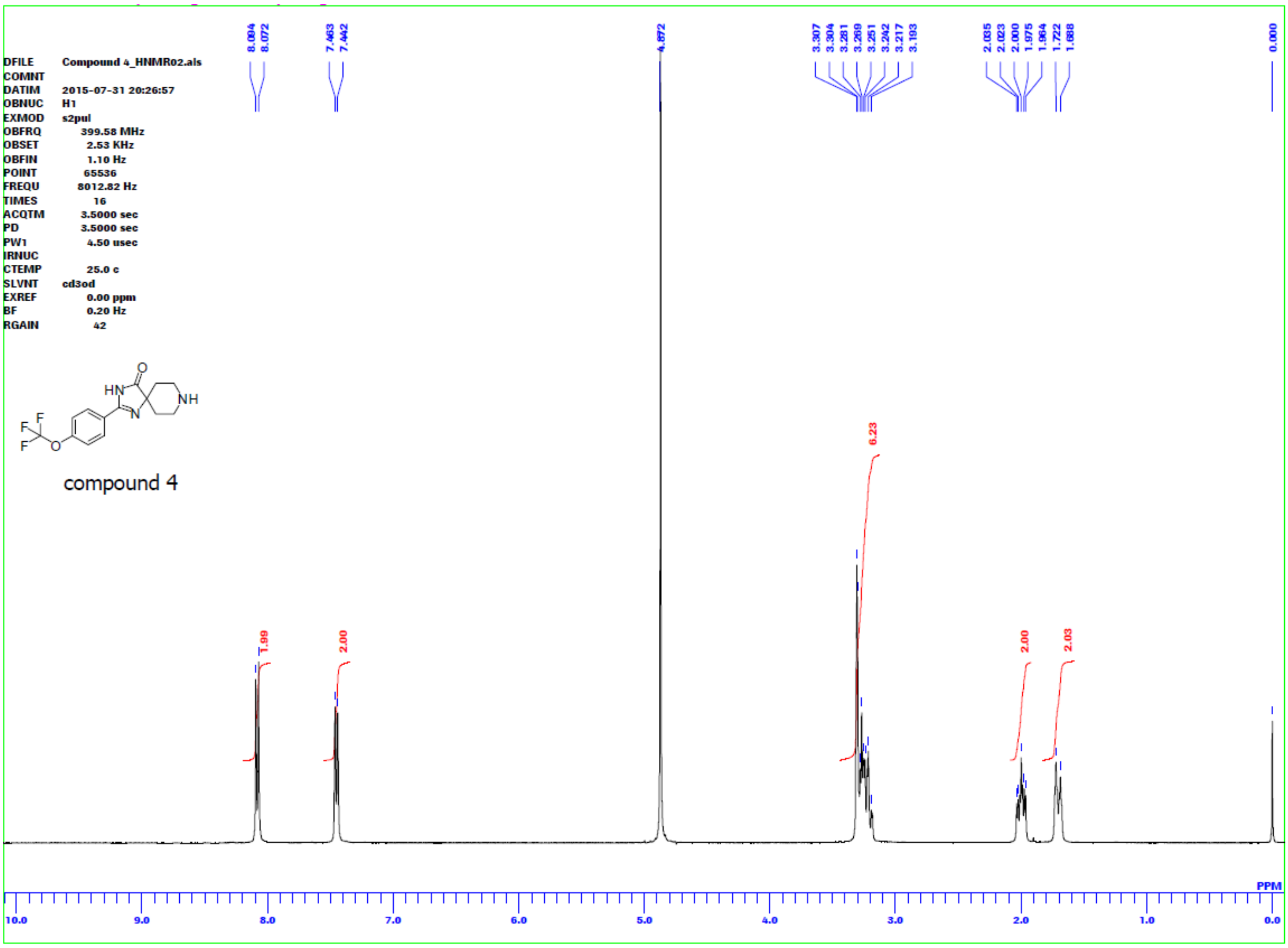

Supplementary Figure 10. ^{1}**H-NMR (400 MHz,** CD_3OD**) spectrum of compound 4.**

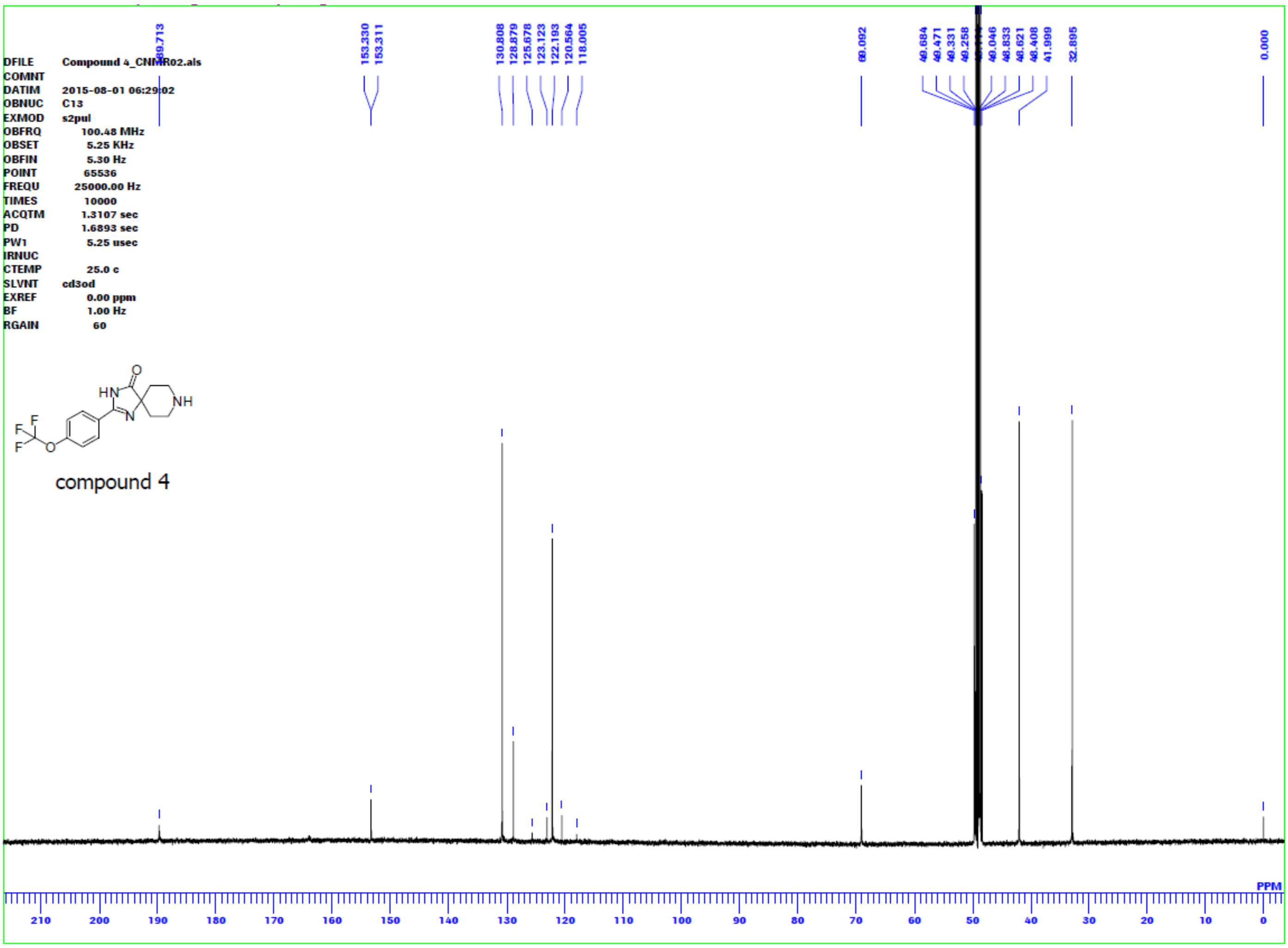

Supplementary Figure 11. ^{13}C-NMR (100 MHz, CD_3OD) spectrum of compound 4.

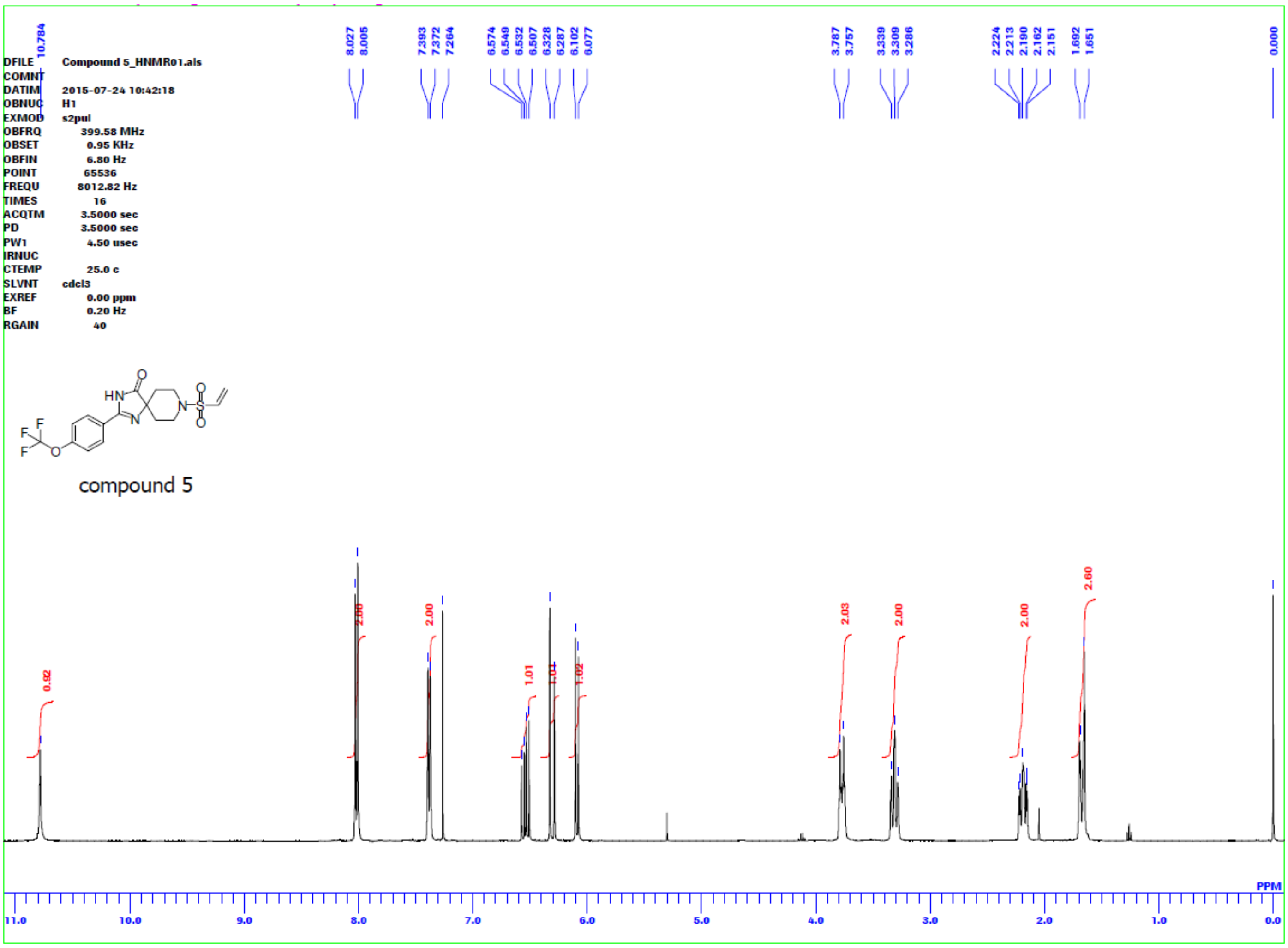

Supplementary Figure 12. ^{1}H-NMR (400 MHz, $CDCl_3$) spectrum of compound 5.

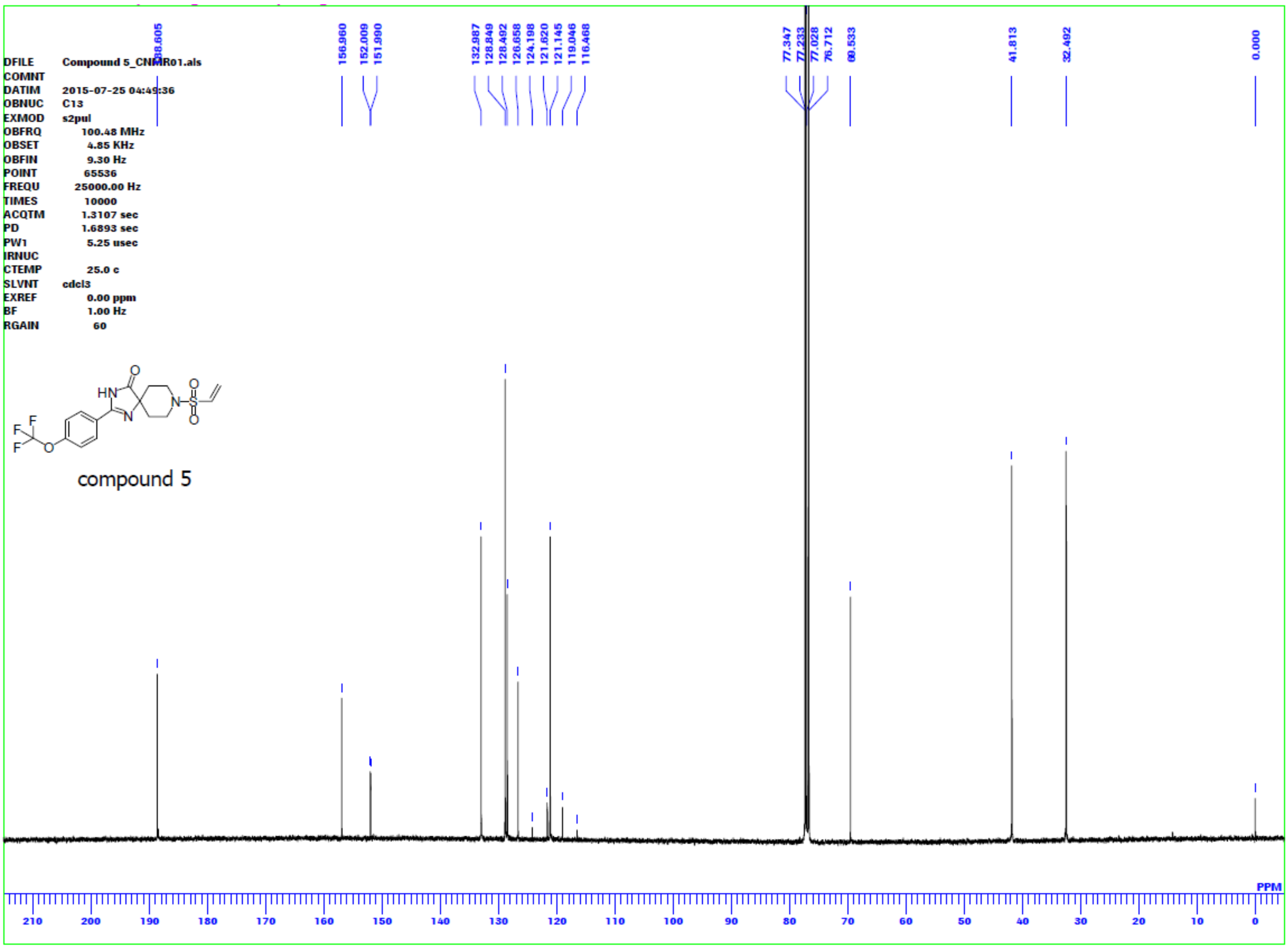

Supplementary Figure 13. ^{13}**C-NMR (100 MHz,** $CDCl_3$**) spectrum of compound 5.**

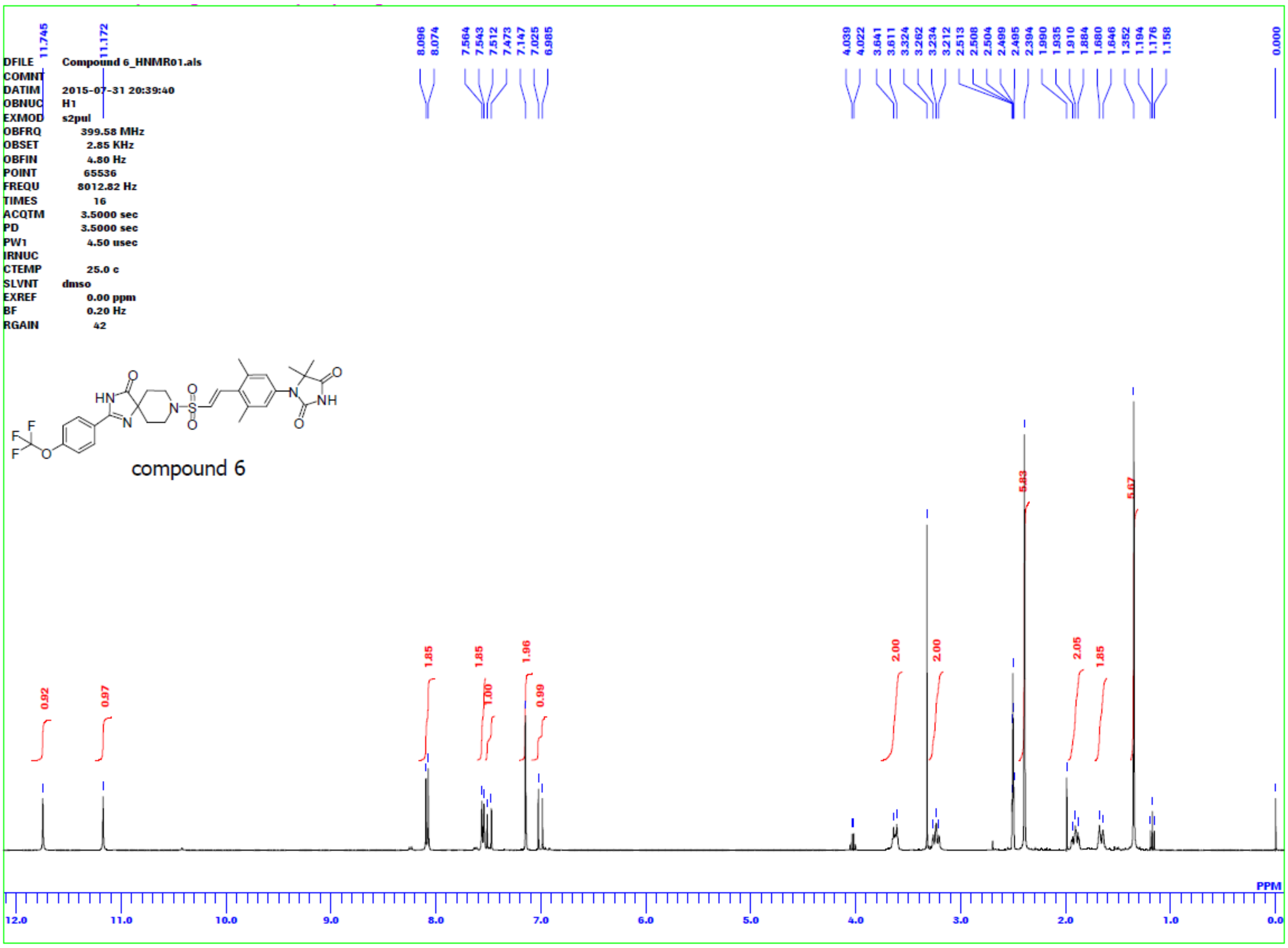

Supplementary Figure 14. ^{1}H-NMR (400 MHz, DMSO-d_6) spectrum of compound 6.

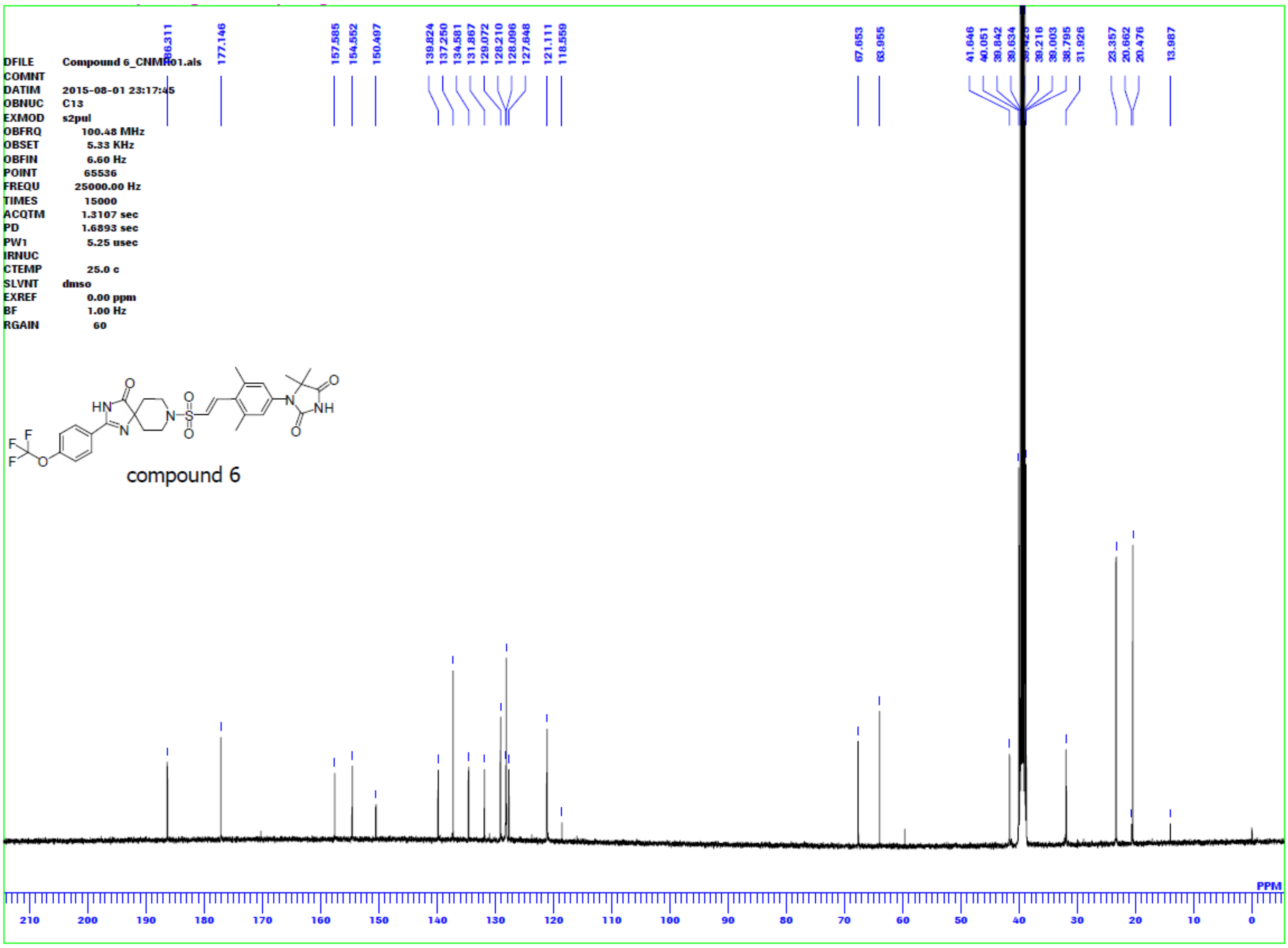

Supplementary Figure 15. ^{13}C-NMR (100 MHz, DMSO-d_6) spectrum of compound 6.

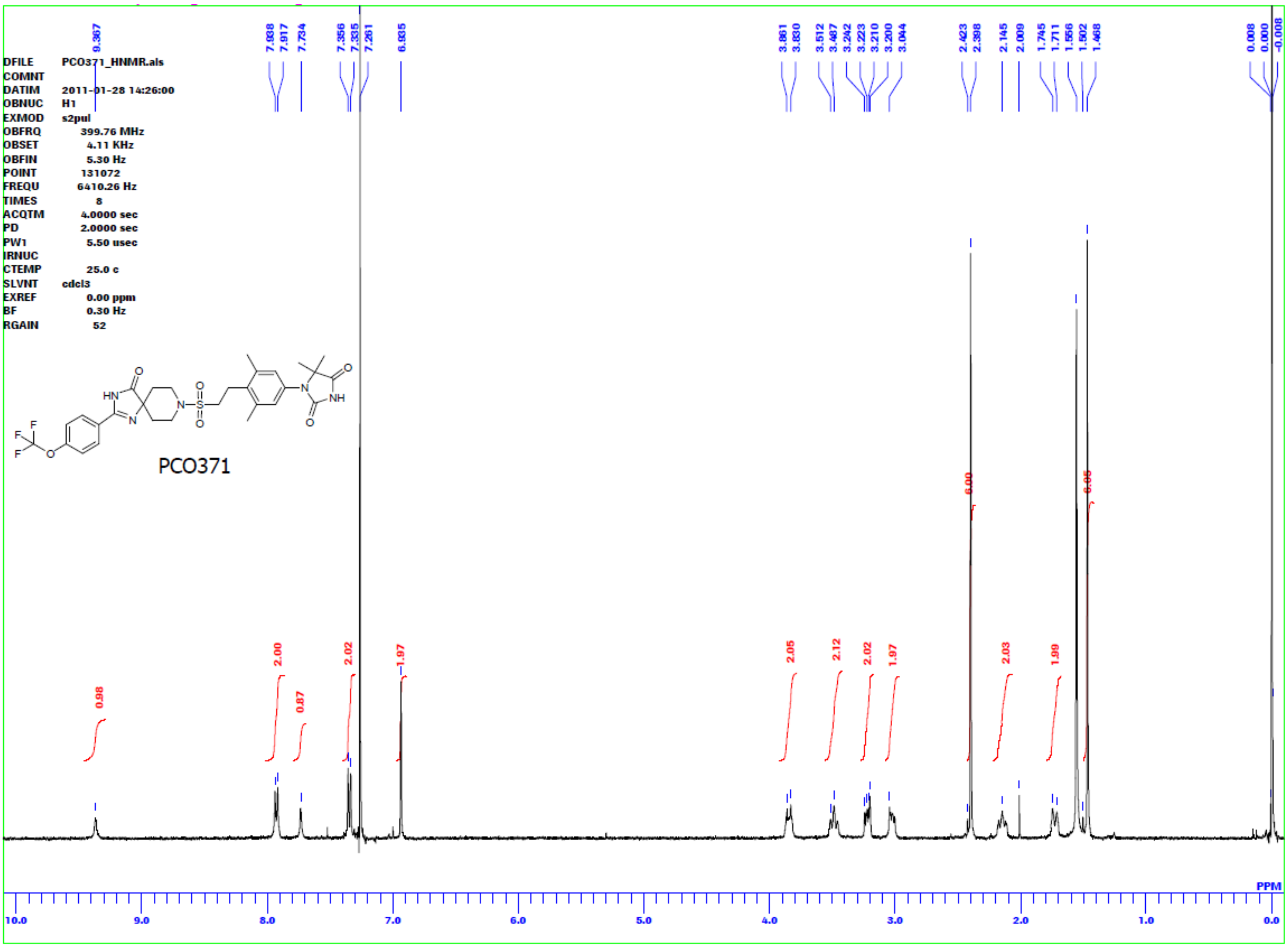

Supplementary Figure 16. ^{1}**H-NMR (400 MHz,** $CDCl_3$**) spectrum of PCO371.**

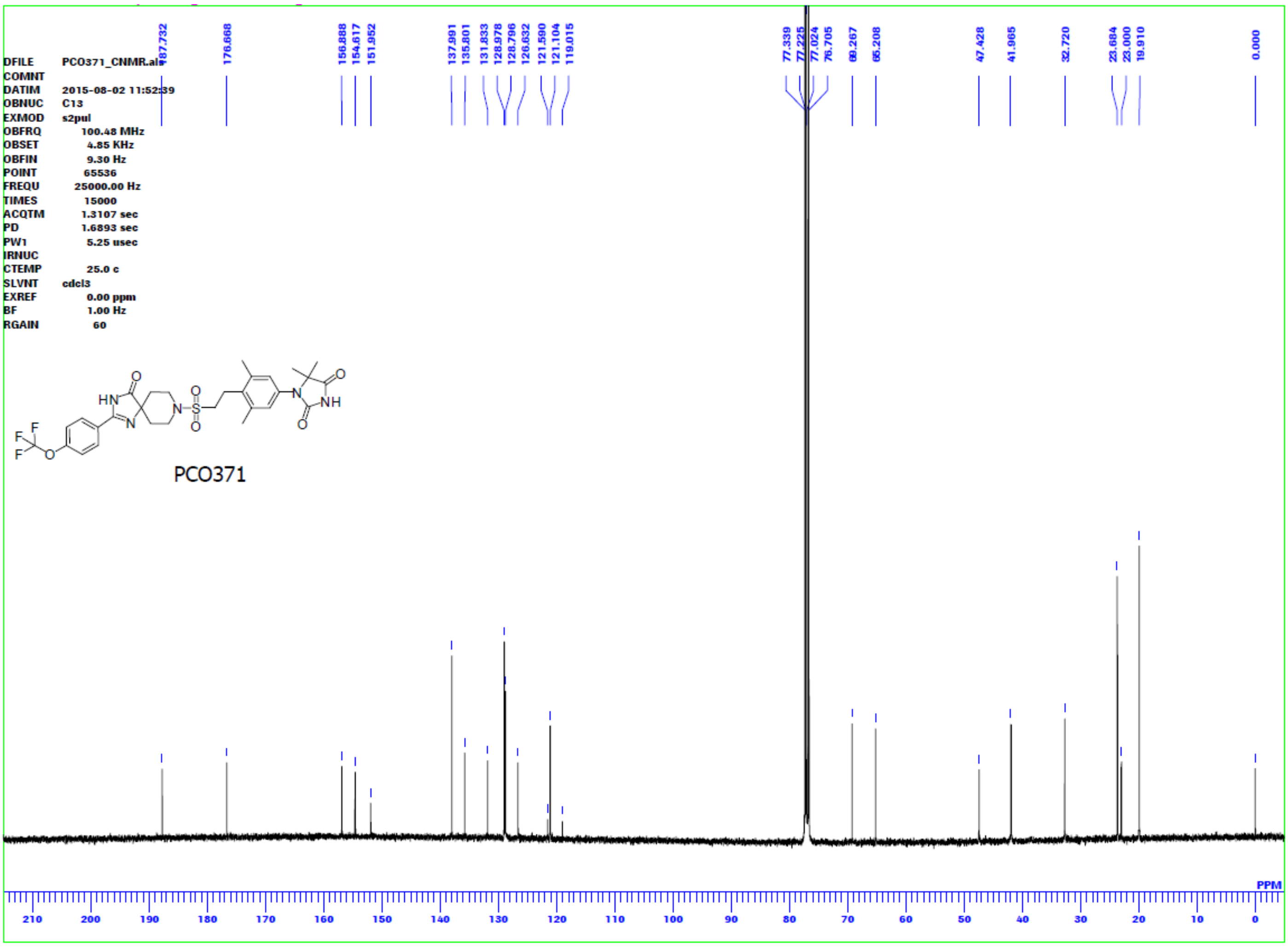

Supplementary Figure 17. ^{13}**C-NMR (100 MHz, CDCl**$_3$**) spectrum of PCO371.**

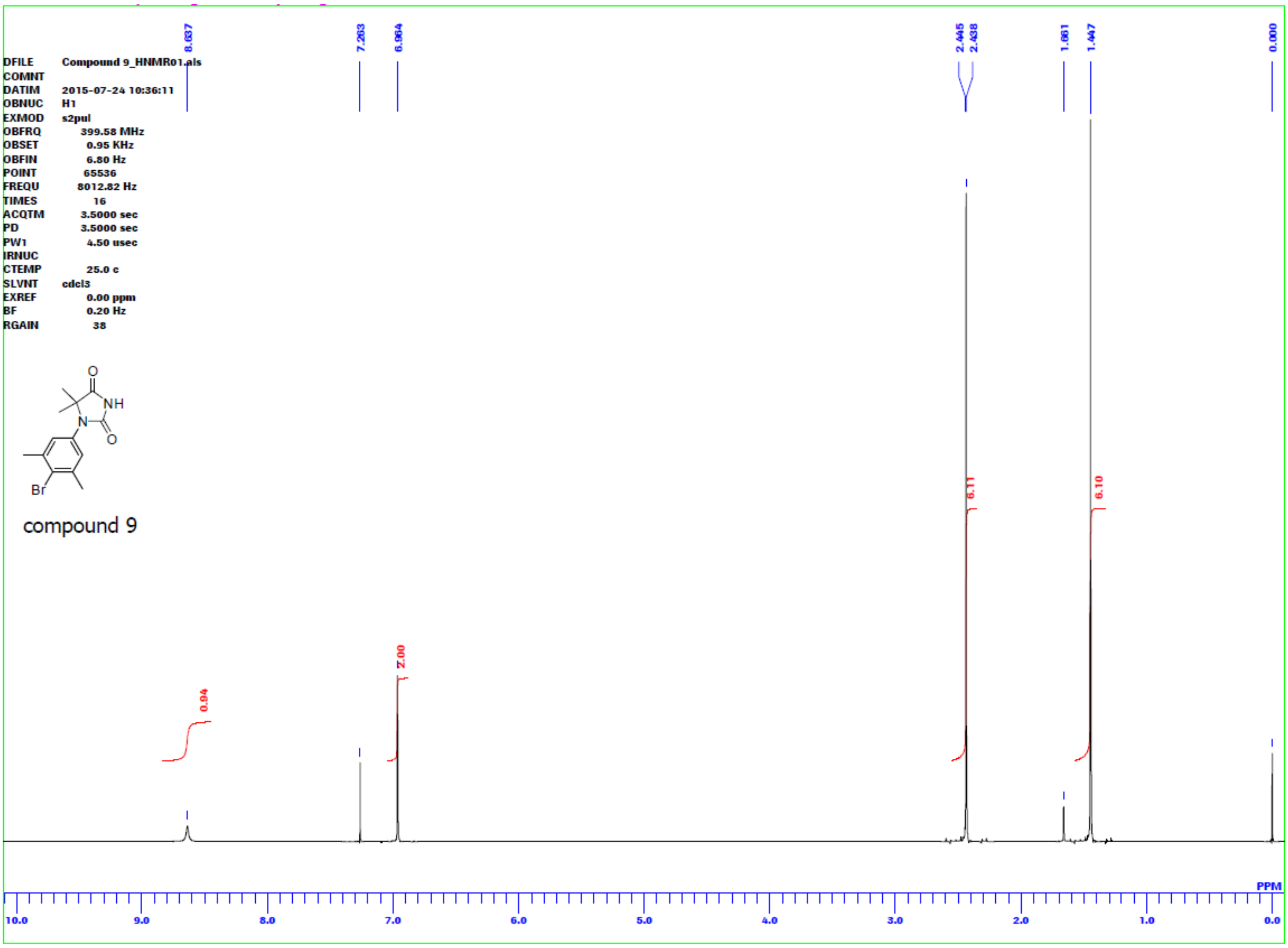

Supplementary Figure 18. ^{1}**H-NMR (400 MHz,** $CDCl_3$**) spectrum of compound 9.**

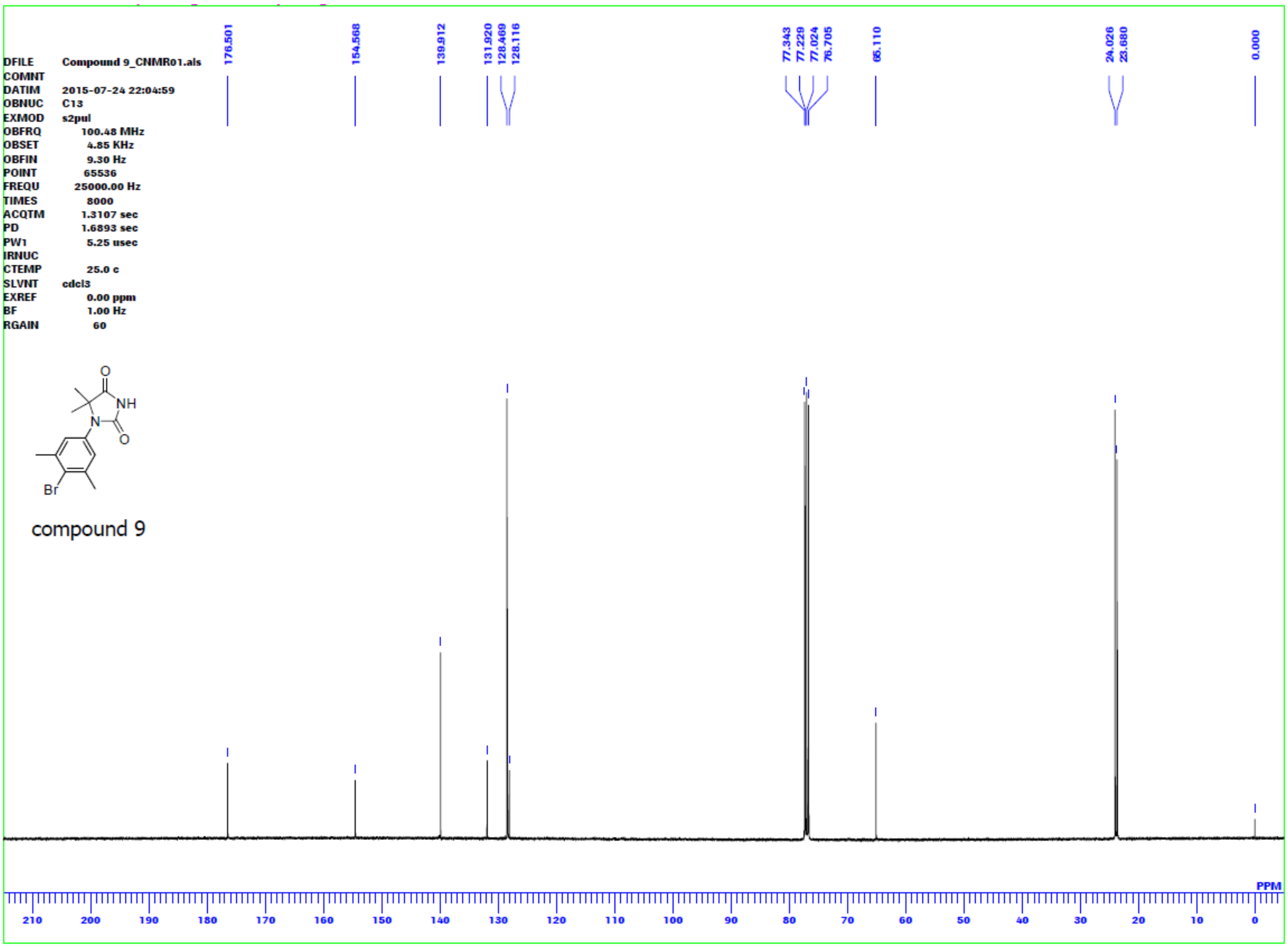

Supplementary Figure 19. ^{13}C-NMR (100 MHz, $CDCl_3$) spectrum of compound 9.

Supplementary Table 1. Agonistic and antagonistic effects of PCO371 on class B GPCRs

GPCR	Agonist effect				Antagonist effect			
	Control agonist		PCO371 (% of control agonist)		Control antagonist		PCO371 (% inhibition of control agonist)	
		EC_{50} (nmol L^{-1})	1 μmol L^{-1}	10 μmol L^{-1}		IC_{50} (nmol L^{-1})	1 μmol L^{-1}	10 μmol L^{-1}
**CGRP**	hCGRPalpha	0.18	<25	<25	hCGRPalpha(8-37)	12	<25	<25
**Calcitonin**	human calcitonin	5.6	<25	<25	salmon calcitonin 8-32	26	<25	<25
**CRF1**	ovine CRF	11, 12	<25	<25	astressin	8.5 ,5.1	<25	<25
**CRF2**	human CRF	14	<25	<25	astressin	14	<25	<25
**GLP-1**	GLP-1-(7-34)	0.075, 0.13	<25	<25	excendin-3(9-39)	8	<25	<25
**GLP-2**	GLP-2-(1-34)	0.077	<25	<25	-	n.d.	<25	<25
**Glucagon**	glucagon	0.092	<25	<25	L168,049	12,000	<25	<25
**Secretin**	human secretin	0.075	<25	<25	-	n.d.	<25	<25
**GHRH**	human GHRF(1-29)	0.95	<25	<25	[N-Acetyl-Tyr1,D-Arg2]-GHRF	2.5	<25	<25
**PAC1**	PACAP1-38	0.11	<25	<25	PACAP6-38	40	<25	<25
**VPAC1**	VIP	0.36	<25	<25	VIP GRF8-27	23	<25	<25
**VPAC2**	VIP	0.89	<25	<25	VIP6-28	25,000	<25	<25

Results of assays for off-target activity of PCO371 against class B GPCRs. PCO371 (1 and 10 μmolL^{-1}) did not show agonistic or antagonistic activity (defined as >25% activity of positive control) against any of the receptors tested, whereas the corresponding agonist or antagonist for each receptor responded at the EC_{50} or IC_{50} indicated. Antagonistic activity was measured in the presence of the control agonist indicated. NA: not applicable; NE: not evaluated. Data from one experiment.

Supplementary Table 2. Pharmacokinetics profile in OVX rats

	Dose (mg kg^{-1})	Route	T1/2 (hour)	Tmax (hour)	Cmax (ng mL^{-1})	C2min (ng mL^{-1})	AUC (ng h mL^{-1})
**PCO371**	30	p.o.	2.5 ± 0.3	1.4 ± 0.8	95.6 ± 84.4	NE	29,500 ± 17,800
	3	i.v.	2.4 ± 0.2	NE	NE	17,500 ± 2,330	15,500 ± 2,820
	10	i.v.	2.2 ± 0.2	NE	NE	50,100 ± 6,910	48,700 ± 8,620

OVX rats were treated with a single subcutaneous or a single oral administration of PCO371. Blood samples were collected at 2 min (i.v. only), 15, 30 min, and 1, 2, 4, 8, and 24 h, and pharmacokinetics parameters were determined. C2min: Concentration at 2 min after administration. Data are represented as the mean ± s.d. of one experiment (n=10 for 10 mg kg^{-1} PCO371, n=11 for 3 and 30 mg kg^{-1} PCO371). NE: not evaluated.

Supplementary Table 3. Bone histomorphometry of lumbar vertebra (L2) in OVX rats

	Sham	OVX				
			PCO371			PTH(1-34)
	Vehicle	Vehicle	p.o. 30 mg kg^{-1}	i.v. 3 mg kg^{-1}	i.v. 10 mg kg^{-1}	s.c. 0.9 nmol kg^{-1}
**BV/TV** (%)	30.4 ± 1.6	17.5 ± 1.3 #	20.1 ± 1.4	23.2 ± 1.9 *	20.1 ± 1.1	34.6 ± 1.8 ***
**Tb. Th** (μm)	77.5 ± 4.4	61.2 ± 2.4 #	76.0 ± 3.1 *	75.1 ± 4.1 *	79.2 ± 2.7 *	99.4 ± 3.5 ***
**Tb. N** (N per mm)	3.95 ± 0.15	2.85 ± 0.15 #	2.64 ± 0.14	3.07 ± 0.18	2.54 ± 0.11	3.47 ± 0.12 **
**Tb. Sp** (μm)	178 ± 8	301 ± 20 #	315 ± 24	264 ± 24	322 ± 18	193 ± 12 ***
**ObS/BS** (%)	4.05 ± 0.77	15.09 ± 1.09 #	25.14 ± 2.09 ***	14.98 ± 1.07	24.64 ± 2.70 **	17.00 ± 1.65
**N.Ob/BS** (N per mm)	3.67 ± 0.65	11.39 ± 0.78 #	17.55 ± 1.43 **	11.36 ± 0.71	16.09 ± 1.72 *	12.65 ± 1.04
**MAR** (μm per day)	1.48 ± 0.11	2.01 ± 0.03 #	2.28 ± 0.08 **	1.84 ± 0.07	2.22 ± 0.06 *	2.04 ± 0.05
**BFR/BS** (mm^3 per mm^2 per year)	0.029 ± 0.007	0.134 ± 0.012 #	0.285 ± 0.023 ***	0.144 ± 0.013	0.275 ± 0.022 ***	0.224 ± 0.012 ***
**OV/TV** (%)	0.146 ± 0.030	0.433 ± 0.033 #	0.794 ± 0.085 ***	0.465 ± 0.040	0.690 ± 0.076 *	0.662 ± 0.055 *
**ES/BS** (%)	14.5 ± 2.0	28.3 ± 2.4 #	39.7 ± 1.6 ***	27.6 ± 2.5	33.0 ± 1.6	20.5 ± 1.6 *
**OcS/BS** (%)	4.28 ± 0.51	8.79 ± 0.95 #	13.60 ± 0.69 ***	8.49 ± 0.79	11.69 ± 0.70 *	6.13 ± 0.58 *
**N.Oc/BS** (N per mm)	1.59 ± 0.22	2.92 ± 0.29 #	4.30 ± 0.27 ***	2.55 ± 0.22	3.60 ± 0.20	1.87 ± 0.19 *
**BRs.R** (mm^2 per mm^2 per year)	0.011 ± 0.002	0.036 ± 0.004 #	0.057 ± 0.006 *	0.039 ± 0.005	0.071 ± 0.008 ***	0.068 ± 0.005 ***

Effects of PCO371 and hPTH(1–34) on bone histomorphometry parameters of the cancellous bone of lumbar vertebra (L1) in OVX rats. BV/TV = Bone volume/Tissue volume. Tb.Th = Trabecular thickness. Tb.N = Trabecular number. Tb.Sp = Trabecular separation. Ob.S/BS = Osteoblast surface/Bone surface. N.Ob/BS = Number of osteoblasts/Bone surface. MAR = Mineral apposition rate. BFR/BS = Bone formation rate/Bone surface. OV/TV = Osteoid volume/Tissue volume. ES/BS = Eroded surface/Bone surface. Oc.S/BS = Osteoclast surface/Bone surface. N.Oc/BS = Number of osteoclasts/Bone surface. PCO371 30 mg kg^{-1} (p.o.) and 10 mg kg^{-1} (i.v.) are exposure-matched dose settings. Data are represented as the mean ± s.e.m. (n=9–12) of one experiment. Student's t-test was used to compare the sham and the OVX control groups; #: P<0.05. Parametric Dunnett's test was used to compare each OVX treated group with the OVX control group; *: P<0.05, **: P<0.01, ***: P<0.001.

Supplementary Table 4. Pharmacokinetics profile in TPTX rats

	Route	Dose	$T_{1/2}$ (h)	T_{max} (h)	C_{max} (ng mL^{-1})	AUC (ng h mL^{-1})
**hPTH(1-84) (nmol kg^{-1})**	s.c.	30	0.2 ± 0.1	0.2 ± 0.2	3.42 ± 1.10	1.80 ± 0.78
		100	1.7 ± 2.2	0.1 ± 0.1	8.67 ± 1.97	8.55 ± 4.63
		300	1.1 ± 0.9	0.1 ± 0.1	39.7 ± 10.3	21.0 ± 5.58
**PCO371 (mg kg^{-1})**	p.o.	2	6.9 ± 2.3	1.4 ± 1.0	95.6 ± 84.4	464 ± 125
		6	4.9 ± 0.6	1.3 ± 0.6	216 ± 103	1,590 ± 890
		18	4.7 ± 0.7	3.7 ± 3.8	1,630 ± 1,740	9,210 ± 8,080

Pharmacokinetic parameters of PCO371 and hPTH(1–84) in TPTX rats. Rats were treated with a single subcutaneous injection of hPTH(1–84) or a single oral administration of PCO371. Blood samples were collected at 3, 10, 15, 30, and 45 min and at 1, 2, and 4 h for hPTH(1–84) or at 15 and 30 min, and 1, 2, 4, 8, and 24 h for PCO371, and pharmacokinetics parameters were determined. Data are represented as the mean ± s.d. of one experiment (*n*=3 for PCO371, *n*=5 for hPTH(1–84)).
